# Susceptibility to COVID-19 Scams: Does Age Matter?

**DOI:** 10.1093/geroni/igab046.2837

**Published:** 2021-12-17

**Authors:** Julia Nolte, Yaniv Hanoch, Stacey Wood, David Hengerer

**Affiliations:** 1 Cornell University, Ithaca, New York, United States; 2 University of Southampton, Southampton, England, United Kingdom; 3 Scripps College, Claremont, California, United States; 4 Claremont Graduate University, Claremont, California, United States

## Abstract

The COVID-19 pandemic has lead to a worldwide surge in COVID-19 related mass marketing scams. While COVID-19 poses higher health risks for older adults, it is unknown whether older adults are also facing higher financial risks as a result of such scams. Thus, the present study examined age differences in vulnerability to COVID-19 scams and factors that might help explain them. In June 2020, 68 younger (18 – 40 years, M = 25.67, SD = 5.93), 79 middle-aged (41 – 64 years, M = 49.86, SD = 7.20), and 63 older adults (65 – 84 years, M = 69.87, SD = 4.50) were recruited through Prolific. Participants responded to five COVID-19 solicitations, psychological measures, and demographic questions. Across solicitations, older adults perceived COVID-19 solicitations to offer significantly fewer benefits than both younger and middle-aged adults did. However, age groups neither differed in their perception of the solicitations’ risks and genuineness nor in their willingness to act in response to COVID-19 solicitations. Overall, intentions to respond to COVID-19 solicitations were positively predicted by higher levels of educational attainment, a previous history of fraud victimization, and higher levels of positive urgency. As expected, stronger genuineness and benefit perceptions positively predicted action intentions, whereas stronger risk perceptions negatively predicted action intentions. Older adults did not exhibit greater vulnerability to COVID-19 solicitations: If anything, they were more skeptical of the benefits associated with these solicitations. Irrespective of age, risk, benefit, and genuineness perceptions were the key factors associated with intention to respond to solicitations.

